# 

**Published:** 1907-12-15

**Authors:** 


### ANNOUNCEMENTS.

Dr. J. P. Buckley has accepted the editorship of the *Dental Digest*, published by Dr. J. N. Crouse, of Chicago. Dr. Buckley is well prepared for this work. He is a forcible and competent writer, and a teacher in the Chicago Dental College. He is young and ambitious, and we confidently expect that he will add materially to the quality of our journal literature. We most cordially welcome him to the work, and hope he shall succeed as well in this effort as he has in his professional and teaching careers.

West Virginia State Dental Society. The annual meeting of the society was held in Parkersburg October 9th, 1907. Dr. H. H. Harrison, of Wheeling, was elected President, and Dr. F. L. Wright, of Wheeling, Secretary.

Institute of Dental Pedagogics. The annual meeting of this society will be held December 31st, 1907, and January 1st and 2nd, 1908, at New Orleans, La. An exceptionally good program has been provided, and the dentists of New Orleans will certainly provide a good time for all who may attend. All college teachers are especially urged to attend, and any dentist who may be interested in teaching methods will be instructed, and gladly welcomed.

The St. Louis Dental Society will hold a meeting in January, at which Dr. E. C. Kirk will present an address on the life and work of Professor W. D. Miller. The occasion will doubtless be one of great interest.

The St. Louis Society of Dental Science, Annual Meeting.—The St. Louis Society of Dental Science will hold its annual meeting at THE JEFFERSON HOTEL, 2:30 P. M., January 21, 1908.

Lecture on "THE LIFE WORK OF PROF. MILLER," by Edward C. Kirk, D. D. S., Sc. D., Philadelphia. Discussion opened by Dr. N. S. Hoff, Ann Arbor, Mich., and Dr. Louis P. Bethel, Columbus, Ohio.

The annual banquet will be given at 7 P. M., of the same day in honor of Prof. Edward C. Kirk, Dean of the Dental Department, University of Pennsylvania and Editor of "*The Dental Cosmos*." The speakers will be: Rev. Dr. Henry Stiles Bradley, Pastor St. Johns M. E. Church, St. Louis; Hon. Arthur W. Sager, Circuit Attorney, St. Louis; Dr. Louis P. Bethel, Edtor "*The Dental Summary*," Columbus, Ohio; Dr. Nelville S. Hoff, Editor THE DENTAL REGISTER, Ann Arbor, Mich.; Dr. Chas. H. Darby, St. Joseph, Mo.; Dr. F. G. Worthley, Associate Editor "*The Western Dental Journal*," Kansas City; Dr. W. L. Whipple, St. Louis; Dr Burton Lee Thorpe, Associate Editor "*The Dental Brief*," St. Louis, Mo.

The profession are invited to attend both the lecture and banquet. For reservation for same and other information, address Dr. Richard Summa, Oriel Building, St. Louis.

W. L. WHIPPLE,<br>

E. E. HAVERSTICK, } *Executive Committee.*<br>

HERMAN F. CASSELL,

D. O. M. LECRON, *Prest.*<br>

C. O. SIMPSON, *Secy.*

